# Delayed Sternal Closure for High-Risk Cardiac Surgery Patients: Life-Saving Strategy for Improved Outcomes

**DOI:** 10.3390/jcm15020423

**Published:** 2026-01-06

**Authors:** Sahin Iscan, Ertürk Karaağaç, Nuri Utkan Tunca, Hacı Anıl Solak, Hasan İner, Serkan Yazman, Yuksel Besir, Orhan Gökalp, Levent Yılık, Ali Gürbüz

**Affiliations:** Department of Cardiovascular Surgery, Katip Çelebi University Izmir Atatürk Training and Research Hospital, İzmir 35360, Turkey

**Keywords:** delayed sternal closure, bleeding, low cardiac output

## Abstract

**Background/Objectives**: Delayed sternal closure (DSC) is a useful management strategy for complex cardiac interventions. The aim of this study was to investigate the patients who had DSC in our clinic over a 12-year period and to evaluate the postoperative results. **Methods**: A total of 124 DSC patients from a total cardiac surgery practice during a 12-year period (*n* = 6532, 1.8%, between January 2014 and September 2025) were retrospectively analyzed. Preoperative and intraoperative patient characteristics, morbidities, and mortality rates were collected and compared with the group undergoing primary sternal closure (PSC), which were matched with the DSC group in terms of preoperative and intraoperative patient characteristics. **Results**: A total of 124 (1.8%) patients required DSC, and 33.1% of the patients were females. The indications were bleeding (*n* = 81, 65%) and hemodynamic instability (*n* = 43, 35%). Total bypass times, cross-clamp times, and CPB temperature were higher in patients with DSC. A higher rate of inotropic support, intra-aortic balloon pump, extracorporeal lung support, blood transfusion, and bleeding were found in the DSC group. There was no difference in terms of sternal infection rate (2.4%). Intensive care unit stay, hospital stay, and mortality rate were also significantly increased in patients with DSC. Mortality rate in the DSC group was 16.1%. **Conclusions**: Multiple sternum revisions due to bleeding and low cardiac output syndrome may lead to increased mortality in high-risk patients. Planned postponement of sternal closure in these high-risk cardiac surgery patients helps to reduce perioperative morbidity and mortality.

## 1. Introduction

The primary determinants of mortality in cardiovascular surgery include the risk of hemorrhage and low cardiac output syndrome. As the surgical technique becomes more complex and the duration of surgery and cardiopulmonary bypass (CPB) extends, the risk of bleeding, morbidity, and mortality rates rises accordingly [1]. Consequently, certain cardiovascular procedures carry an increased risk of bleeding starting from the preoperative period due to the nature of surgical intervention. CPB remains the leading factor contributing to increased bleeding risk. Prolonged CPB durations can induce de novo coagulation disorders that were absent preoperatively, thereby escalating the risk of postoperative hemorrhage and potentially necessitating repeated surgical re-explorations [2,3]. In this context, the most useful technique that we can apply is mediastinal packing and the delayed sternal closure (DSC) technique to avoid repeated revisions. The aim of this study is to retrospectively evaluate patients who underwent DSC at our institution over the past 12 years, identify predictable preoperative and perioperative risk factors, and analyze the associated postoperative outcomes.

## 2. Materials and Methods

This study was designed retrospectively after the approval of the local ethical committee and was conducted in accordance with the principles set forth in the Helsinki Declaration, as revised in 2024. All patients undergoing DSC after open adult cardiac surgery between January 2014 and September 2025 in our clinic were investigated. DSC was defined as leaving the sternum open with mediastinal packing at the end of the operation. Out of 6532 patients who underwent cardiac surgery during this period, 124 required DSC, resulting in an incidence of 1.8%. Patient demographics, preoperative characteristics, operative data, and perioperative complications were statistically investigated. The DSC group was compared with the primary sternal closure group (PSC), consisting of patients who underwent similar surgical interventions with sternal closure and were matched for preoperative and intraoperative patient characteristics. To accurately compare the revision rate between the groups, PSC patients who required re-intervention were also included in the study. DSC decision was made at the end of the surgical intervention. The factors influencing DSC decision were as follows: complex cardiac procedures; severe systolic/diastolic dysfunction and hemodynamic instability at the end of the operation, necessitating maximal inotropic support; prolonged cross-clamp and cardiopulmonary bypass times; coagulopathy; intractable bleeding (exceeded 50 mL within 30 min), a requirement for multiple blood products transfusion; and anticipated high likelihood of needing revision surgery during intensive care unit follow-up. At the end of the operations, sponges were placed to compress the mediastinal tissues. Subsequently, the sternum was left open, and the skin was closed with continuous Prolene sutures. All patients were transferred to the intensive care unit, where they were ventilated and sedated until their chest closure. Sternal closure time was determined based on the patient’s hemodynamic stability and drainage. In the following days, once the bleeding stabilized or the need for ventricular support decreased, patients were returned to the operating room for a definitive sternal closure.

The mean, standard deviation, median, minimum, maximum, frequency, and percentage values were used for descriptive statistics of the data. The Kolmogorov–Smirnov and Shapiro–Wilk test were used for measuring the distribution of the variables. An independent sample *t*-test was used for analysis of the quantitative independent data with a normal distribution. Mann–Whitney U Test was used for analysis of the quantitative independent data without a normal distribution. Chi-square test was used for analysis of the qualitative independent data; if chi-square test conditions were not met, the Fisher Test was used. All statistical computations were performed using SPSS version 27.0 (IBM Corp., Armonk, NY, USA).

## 3. Results

**Figure 1:**
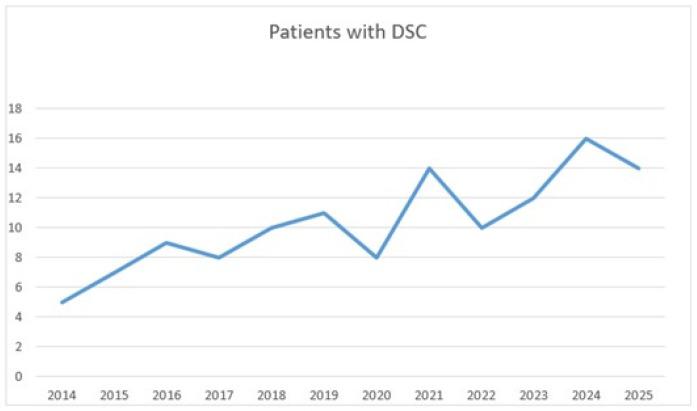
Use of DSC technique between 2014 and 2025.

Patient demographics and co-morbidities are presented in Table 1. A total of 124 (1.8%) patients required DSC in a 12-year period at our clinic (Figure 1). Overall, the mean age was 58.4 ± 13.9 years, and 33.1% of the patients were females. Cardiac operations and cardioplegics used are detailed in Table 2. The most frequent indication was severe coagulopathy in 81 patients (65%), followed by hemodynamic instability in 43 patients (35%), and combined hemodynamic instability and severe coagulopathy in 17 patients (13%). There was no difference among age, gender, co-morbidities, ejection fractions, preoperative creatinine values, performed operations, emergency operations, and redo-surgeries between DSC and PSC groups (*p* > 0.05). There was a significant difference between the two groups in terms of Euroscore II risk stratifications, which were 2.56 ± 2.04 in the PSC group and 3.44 ± 3.23, *p* = 0.046 in the DSC group.

Total bypass times and cross-clamp times were longer in patients with DSC (152.8 ± 63.1 and 95.9 ± 43.5 min) compared with patients in the PSC group (96.8 ± 38.1 and 64.1 ± 26.0 min) (*p* ≤ 0.001). Cardiopulmonary bypass temperature was significantly decreased in patients with DSC (27.6 ± 5.1 °C and 29.5 ± 2.7 °C, *p* ≤ 0.001). Higher rates of inotropic support, intra-aortic balloon pump, and intraoperative extracorporeal lung support (ECLS) were found in the DSC group (*p* ≤ 0.001, *p* ≤ 0.001, and *p* = 0.004) (Table 3).

There was a significant difference in terms of blood transfusion and bleeding rate between the DSC and PSC groups. A total of 1.9 ± 0.9 units of red blood cell suspensions were transfused to the DSC group, and 1.2 ± 0.4 units of red blood cell suspensions were transfused to the PSC group (*p* ≤ 0.001). First day drainage amount was 641.9 ± 192.8 mL in the DSC group versus 449.3 ± 165.3 mL in the PSC group (*p* = 0.006); the first revision operation rate was 19% (*n* = 24), and the second revision operation rate was 9% (*n* = 12). Intraoperative hemofiltration rate was 32.3% (*n* = 40) in the DSC group versus 9.9% (*n* = 15) in the PSC group, and the hemofiltration amount was 1840.0 ± 694.2 mL in the DSC group versus 506.7 ± 153.4 mL in the PSC group (*p* ≤ 0.001) (Table 3).

There was no difference in terms of sternal infection rate (in the DSC group, 2.4%, *n* = 3) between the groups. But intensive care unit stay (11.3 ± 17.0 days), hospital stay (16.3 ± 18.2 days), and mortality rate were found in 16.1% (*n* = 20) patients with DSC compared to the PSC group.

## 4. Discussion

Delayed incisional closure is a critical strategy for patients with high postoperative risk and for surgeons. Elective or emergency surgical interventions in the abdominal and thoracic cavities carry the risk of ischemia–reperfusion injury, organ edema, and hemorrhage. These problems can lead to abdominal or mediastinal tamponade and often necessitate repeated re-exploration surgeries. In some instances, abdominal organs and the myocardium require additional time to recover and to reach full functional capacity. Delayed closure techniques provide patients with the necessary time to recover and decrease the patient’s mortality, which is higher in this high-risk group of patients. In general surgical and heart surgery practice, it is often safely implemented with an effective decrease in mortality, and without high infection rates, which are not as high as feared for high-risk patients in high-volume surgical centers. Delayed closure is associated with a 15.8% mortality in patients with emergency general surgery, and there was no significant difference observed in terms of infection rates for surgical sites and sepsis incidence [4]. Another study involving patients with isolated mesenteric ischemia reported a mortality rate of 38.9%; although higher than total general surgery cohorts, delayed closure of the incision remained the most efficient strategy for this group [5]. Particularly for patients with trauma and organ ischemia, there is a higher risk of reperfusion, which causes tissue-edema, as it is in cardiac surgical practice. Consequently, there is also a consensus that an open abdomen approach can improve patient outcomes in high-risk general surgery patients [6].

Complex cardiac surgical operations also carry a high risk of morbidity and mortality due to increased surgical risk factors related to the surgical technique that is used. The problems underlying this morbidity and mortality are usually due to the prolonged CPB and myocardial ischemia. In experienced cardiac surgery clinics, taking some precautions in advance to reduce the risk of revision surgery and mortality increases the survival of the patients. DSC, combined with mediastinal packing, is a highly effective surgical technique for managing early postoperative coagulopathy and low cardiac output syndrome following prolonged CPB [7,8,9,10].

Since its introduction in the 1980s, the utilization of DSC has grown, with reported incidence ranging from 1.2% to 4.2% depending on the procedure. For instance, Boken et al. reported a 3.5% incidence of DSC over a 6-year period in a cohort of 6041 patients [11]. Lin et al. examined 704 patients who underwent surgery for type-A aortic dissection over a 14-year period, and they reported the DSC rate as 15.5% [2]. DSC is also an effective and reliable option for pediatric patients. Kundan et al. investigated pediatric cardiac surgery patients, and they showed that the incidence of DSC for pediatric patients in 2011 was 10.42% (*n* = 220) [12]. In our 12-year study of 6532 patients who underwent cardiac surgery in our clinic, 124 individuals underwent the DSC procedure, and they represented an incidence of 1.8% for the general cardiac surgical population.

The primary finding of our study was the indications of DSC; a total of 81 (65%) of the patients underwent a DSC procedure due to coagulation problems, and 43 (%35) of the patients required it due to low cardiac output syndrome. The most important indications for DSC are intraoperative coagulation system disorders and low cardiac output due to myocardial damage and edema after the reperfusion period [13]. Key intraoperative risk factors include cross-clamp time (CCT), CPB time, hypothermia, and myocardial protection methods. These factors can trigger coagulopathy, myocardial contractility problems, and edema after cardiac surgery. As CPB time prolongs, the amount of applied anticoagulation and the consumption of coagulation factors, together with platelets, increase, and as hypothermia progresses, especially when the temperature drops below 28 °C, problems in the coagulation cascade increase, further escalating bleeding risk [14]. In patients with aortic dissection, where these factors are most frequently combined, the increased consumption of platelets and coagulation factors due to acute thrombotic activity in the false lumen significantly increases the risk of bleeding after CPB. Prolonged CPB times, myocardial ischemia time, and increased inflammation also cause the same problems [15,16].

The recent literature indicates an increasing trend in the use of DSC [2,11]. This rise is attributed to several factors: an increase in emergency surgical interventions, longer CPB durations due to the development of cardioplegia techniques, increased risk of bleeding with growing access to right and left heart support systems, and the increased ability to perform redo surgeries with the development of surgical techniques over the years. These changes have made the DSC technique widespread over the years, especially in clinics with dense patient populations. Our clinical experience mirrors these findings. When adult and pediatric cardiac surgery studies in the literature, in which the DSC technique was applied, were examined, it was seen that there were complicated cases where CPB times varied between 60 and 270 min [11,13]. In our study, CPB time was determined as 152.8 ± 63.1 min and CPB temperature as 27.6 ± 5.1 °C in the DSC group. Over the last five years, in our practice, there was a shift; DSC is now more frequently indicated for coagulopathy following high-risk operations rather than for low cardiac output. The diversification of cardioplegia options, particularly single-dose solutions like del Nido and Bretschneider solutions, which facilitate longer, more complex cases, has contributed to this shift [17,18,19]. Furthermore, growing clinical experience has increased surgeons’ propensity to opt for DSC to prevent complications such as hemopericardium, organ malperfusion, and the need for excessive blood transfusion. In our population, the rate of using del Nido and Bretschneider cardioplegia in the DSC group was 57.8%, and the number of patients in whom the DSC technique was used due to coagulation problems was 81 (65%). The amount of red blood cell transfusion was 1.9 ± 0.9 unit and the platelet transfusion rate was 2.0 ± 0.9 units, which were statistically significantly higher in the DSC group.

Due to patients with postoperative low cardiac output, an increase in revision rates has become inevitable with the usage of extracorporeal lung support systems, which are applied to the right and left ventricles. In our study, inotropic therapy, IABP, and ECLS served as a vital supportive approach for these patients, and usage rates of these devices were also found to be significantly higher in DSC patients (*p* < 0.05). ECLS systems, especially with the effect of oxygenators, cause the loss of platelets and coagulation factors. But these systems are inevitable techniques with high bleeding risk and revision rates due to the necessity of anticoagulation. IABP may also cause increased platelet loss in the long term. In these patients, DSC stands out as a useful supportive approach in the first postoperative days to avoid the risk of bleeding and revision rate, until the recovery of myocardial functions.

Sternal infection rates are one of the major concerns following DSC. However, many studies report no difference between DSC and PSC groups in the literature [20]. In their study on patients who underwent acute type A aortic dissection repair, Lin et al. found the deep sternal wound infection rate to be 4.6% in the DSC group and 2.7% in the PSC group, which is not statistically significant [2]. Similarly, Boeken et al. reported that the deep sternal wound infection rate after DSC was 5.3% (*n* = 10 patients) [11]. In the study by Stulak et al., in which they examined 364 patients who underwent ventricular assist device implantation, DSC was applied to 184 (51%) patients [21]. In this patient group, where the indication for DSC was 84% due to coagulopathy, the rates of mediastinitis, pocket infection, and drive-line infection were found to be similar compared to the PSC group. In our study, the median DSC duration was 1.0 (1.2 ± 0.5) days, and the mediastinal infection rate was 2.4% in the DSC group, which did not differ from the PSC group and from the literature. We believe that this similarity in the infection rates in both of the groups is due to the prevention of repeated sternotomies and early stabilization of patients in the postoperative period.

This study has several limitations, including its single-center retrospective design. Multicenter studies are required to obtain more comprehensive validation. Within our institution, the decision for DSC was based on a collaborative consensus in the operating room, particularly for redo or complicated surgeries, cases with severe hypothermia, or prolonged CPB. Ultimately, the final decision rested with the lead surgeon.

## 5. Conclusions

As a result, in this study, we aimed to present our DSC experiences over a 12-year period and our clinical tendency to patients for high revision risk. We conclude that planned postponement of sternal closure leads to better outcomes than emergent reopening or repeated sternotomies, with the benefits of decreased blood loss, decreased blood transfusion, stable hemodynamics, preserved peripheral organ perfusion, and stabilized inflammatory load. The DSC technique is a useful technique, which can be clearly seen with the increasing clinical experience in high-risk patients, who would face a higher risk of mortality when DSC is not used, and we believe that its use should not be avoided but supported with an international consensus paper; short-term postoperative risks are not different from those in patients undergoing primary sternal closure.

## Figures and Tables

**Table 1:** Patients’ demographics.

	PSC (*n* = 151)		DSC (*n* = 124)		*p*
	Med. ± sd/*n*	%	Med. ± sd/*n*	%	
Age	55.6 ± 12.8		58.4 ± 13.9		0.054 ^m^
Gender	Female: 55	36.4%	Female: 41	33.1%	0.561 X^2^
	Male: 96	63.6%	Male: 83	66.9%	
Hypertension	73	48.3%	56	45.2%	0.599 X^2^
Diabetes mellitus	36	23.8%	33	26.6%	0.598 X^2^
COPD	19	12.6%	10	8.1%	0.225 X^2^
CRD	18	11.9%	10	8.1%	0.293 X^2^
Creatinine (mg/dL)	1.1 ± 0.6		1.2 ± 1.2		0.890 ^m^
	Median: 1.0		Median: 0.9		
	Min–Max: 0.5–4.1		Min–Max: 0.5–12.1		
Emergency surgery	19	12.6%	16	12.9%	0.937 X^2^
Redo surgery	20	13.2	21	16.9	0.393 X^2^
Ejection fraction	55.1 ± 12.1		53.1 ± 12.8		0.169 ^m^
Euroscore II	2.56 ± 2.04		3.44 ± 3.23		0.046 ^m^

COPD: Chronic obstructive pulmonary disease, CRD: Chronic renal diseases. ^m^: Mann–Whitney u test. X^2^: Chi-square test.

**Table 2:** Performed operations and cardioplegia in DSC group.

Operations	DSC (*n*-%)	
CABG	43	34.7%
Bentall operation	42	33.9%
Ascending aortic replacement	26	21.0%
AVR	19	15.3%
MVR	18	14.5%
Arcus replacement	12	9.7%
LVAD	4	3.2%
Lead extraction	2	1.6%
Vena Cava repair	1	0.8%
Pericardiectomy	2	1.6%
VSD repair	1	0.8%
Cardioplegia		
Blood	51	42.2%
Del Nido	47	38.8%
Bretschneider	23	19%

CABG: Coronary artery bypass graft, AVR: Aortic valve replacement, MVR: Mitral valve replacement, LVAD: Left ventricle assist devices, and VSD: Ventricular septal repair.

**Table 3:** Intraoperative and postoperative data.

	PSC (*n* = 151)		DSC (*n* = 124)		*p*
	Med. ± sd/*n*	%	Med. ± sd/*n*	%	
CCT (min)	64.1 ± 26.0		95.9 ± 43.5		<0.001 ^m^
	Median: 59		Median: 92.5		
	Min–Max: 20.0–166.0		Min–Max: 0.0–209.0		
TBT (min)	96.8 ± 38.1		152.8 ± 63.1		<0.001 ^m^
	Median: 87		Median: 140		
	Min–Max: 34.0–240.0		Min–Max: 0.0–366.0		
CPB temperature (°C)	29.5 ± 2.7		27.6 ± 5.1		<0.001 ^m^
Inotropic treatment	26	17.2%	53	42.7%	<0.001 X^2^
IABP	5	3%	18	14.0%	<0.001 X^2^
ECLS	0	0%	8	6%	0.004 X^2^
Red blood cell transfusion (unit)	1.2 ± 0.4		1.9 ± 0.9		<0.001 ^m^
	Median: 1.0		Median: 2.0		
	Min–Max: 1.0–2.0		Min–Max: 1.0–5.0		
Bleeding (mL)	449.3 ± 165.3		641.9 ± 192.8		<0.001 ^m^
	Median: 400.0		Median: 600.0		
	Min–Max: 150.0–1000.0		Min–Max: 250.0–1300.0		
Platelet transfusion	0 ± 0.2		2.0 ± 0.9		<0.001 ^m^
	Median: 1.0		Median: 2.0		
	Min–Max: 0.0–1.0		Min–Max: 1.0–5.0		
Revision rate	I = 10	6.6%	I = 24	19% 9%	<0.001 X^2^
	II = 0	00%	II = 12		
Ultrafiltration	15	9.9%	40	32.3%	
Ultrafiltration amount (mL)	506.7 ± 153.4		1840.0 ± 694.2		<0.001 ^t^
	Median: 500.0		Median: 1900.0		
	Min–Max: 300.0–800.0		Min–Max: 500.0–3500.0		
Median DSC duration			1.2 ± 0.5		
			Median: 1.0		
			Min–Max: 1.0–3.0		
ICU stay (days)	2.2 ± 1.1		11.3 ± 17.0		<0.001 ^m^
	Median: 2.0		Median: 6.0		
	Min–Max: 1.0–11.0		Min–Max: 0.0–143.0		
Hospital stay (days)	6.5 ± 2.7		16.3 ± 18.2		<0.001 ^m^
	Median: 6.0		Median: 10.0		
	Min–Max: 2.0–23.0		Min–Max: 0.0–143.0		
Sternal infection	2	1.3%	3	2.4%	0.499 X^2^
Mortality	7	4.6%	20	16.1%	0.001 X^2^

CCT: Cross-clamp time, TBT: Total bypass time, CPB: Cardiopulmonary bypass, IABP: Intra-aortic balloon pump, ECLS: Extracorporeal lung support, and ICU: Intensive care unit. ^m^: Mann–Whitney u test, X^2^: Chi-square test, and ^t^: Independent sample *t*-test.

## Data Availability

The data presented in this study are available on request from the corresponding author due to ethical reasons.
